# The impact of leader depletion on leader performance: the mediating role of leaders’ trust beliefs and employees’ citizenship behaviors

**DOI:** 10.1038/s41598-022-24882-3

**Published:** 2022-11-30

**Authors:** Tessa Haesevoets, David De Cremer, Leander De Schutter, Marius van Dijke, Henry Robin Young, Hun Whee Lee, Russell Johnson, Jack Ting-Ju Chiang

**Affiliations:** 1 Department of Developmental, Personality and Social Psychology, Ghent University, Henri Dunantlaan 2, B-9000 Ghent, Belgium; 2 NUS Business School, National University of Singapore, Singapore, Singapore; 3 Rotterdam School of Management, Erasmus University Rotterdam, Rotterdam, The Netherlands; 4 Eli Broad School of Business, Michigan State University, East Lansing, USA; 5 Fisher College of Business, The Ohio State University, Columbus, USA; 6 Guanghua School of Management, Peking University, Beijing, China

**Keywords:** Psychology, Human behaviour

## Abstract

The leadership role can be demanding and depleting. Using self-regulation and social exchange theory as a framework, we developed a three-step sequential mediation model that explains how feelings of depletion can degrade leaders’ own performance level, via the reciprocating behavior of their employees. Specifically, we hypothesized that leader depletion is negatively related to their trust beliefs. This lack of trust is expected to be reciprocated by employees in such a way that they display less citizenship behaviors towards their leader. These lowered citizenship behaviors are, in turn, predicted to negatively impact leader performance. Additionally, we hypothesized that these negative effects of feeling depleted are more pronounced for leaders who believe that their willpower is limited. Studies 1 and 2 illustrated that leader depletion indirectly influences their own performance level through leaders’ trust beliefs and employees’ leader-directed citizenship behaviors. Study 3 extended these findings from the inter-individual to the intra-individual level, and demonstrated the predicted moderating role of belief in limited willpower. Together, our studies provide new and useful insights in the broader, more distal implications of leader depletion, which have not yet been considered in existing self-regulation models.

## Introduction

Contemporary organizations are characterized by highly demanding environments. In such environments, members at all levels of the organization may feel that their resources are getting depleted^1,2^. When individuals feel depleted, they find it harder to plan and achieve their goals and, as a result, they may perform less well^3^. Within the literature, these types of depletion effects are commonly referred to as self-regulation failures^4,5^. One type of organization member who is particularly confronted with a demanding workload are organizational leaders. Indeed, those in leadership positions are frequently confronted with challenging and complex problems that put constraints on their finite pool of self-regulatory resources^6–8^, which makes them especially prone to self-regulation failures.

Although being a leader can be a demanding and depleting job, some prior research suggests that those in a leadership position are not always influenced by their decreased mental resources and that their performance level can remain unchanged^9,10^. Despite these insights, we argue that leader depletion can nonetheless harm leader performance, especially so as leaders’ role in their organization is socially embedded and many of their tasks are interdependent in nature and therefore require a certain level of cooperation with others (see^11–13^). Because of this embeddedness, we expect that leader depletion can indirectly harm leaders’ own performance level, and that this will happen through their interactions with their employees. Specifically, we develop the argument that leaders’ attitudes towards their employees as a result of feeling depleted shape a negative exchange relationship in which employees’ reciprocating behaviors will be detrimental to the performance of their leader.

**Figure 1:**
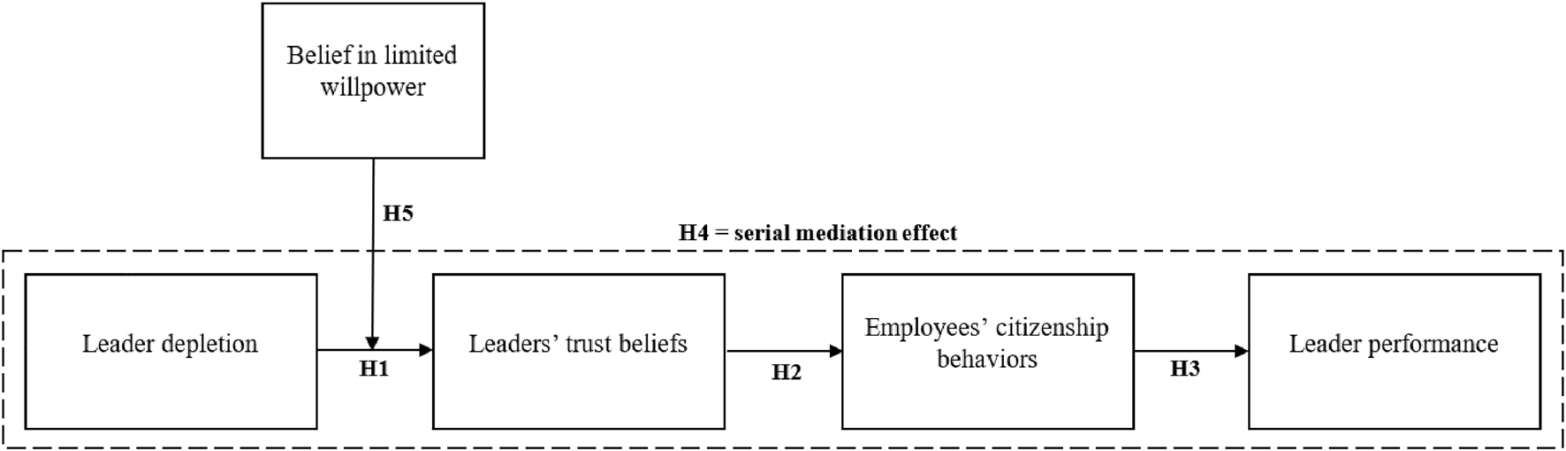
Conceptual model of the effects of leader depletion on leader performance.

In explaining how leader performance can be negatively influenced by leaders’ own depletion level, via the reciprocating behavior of their employees, our model adopts a three-stage process. The first step of our model assumes that feelings of depletion will make leaders less willing to trust others. In a second step of our model, these negative trust beliefs are expected to be reciprocated by employees in such a way that they show decreased organizational citizenship behaviors towards their leader. The third step of our model assumes that, as an important downstream consequence, these lowered citizenship behaviors will undermine the leader’s own work progress. In addition, we also test a potential boundary condition of this model, as not all leaders are expected to be equally affected by a depleted state of mind. In this vein, we envision the predicted negative impact of leader depletion to be more pronounced for leaders who hold the belief that their willpower to exert self-control is a limited resource. Figure 1 displays our conceptual model.

### Theoretical background

Leaders face continuous pressure to uphold their own performance level. In doing so, leaders need to regularly assess whether discrepancies exist between their current level of performance and their expected goals. Self-regulation theories adopt a specific focus on how people can internally manage such a process at work^14^. As soon as an individual sets his or her goals, this internal management process is activated and continued throughout the goal pursuit process^15^. In order for this continuous internal management process to succeed, the individual needs to be able to exercise self-control and withstand distracting urges and temptations that could undermine the goal pursuit process. Of course, for an individual to effectively regulate this goal-pursuit process, he or she needs to have sufficient resources available to draw the necessary energy from. Specifically, if there is a discrepancy between one’s current performance level and one’s desired goals, then more energy needs to be devoted for improving the pursuit of one’s goals. An important assumption of self-regulation models, however, is that individuals can only draw mental energy from a limited and finite pool of resources^4,5^. These models assume that people’s energy levels are limited and, once those resources are depleted, people will feel mentally exhausted, which makes it harder to exert self-control and foster the internal management of one’s goal pursuit. In line with this assumption, a recent study reported that when leaders feel that their resources get depleted their capacity to engage in work is diminished^6^. Besides, it has been found that resource depletion at work also leads to higher levels of conflict at home^16^.

Although organizational leaders are confronted daily with responsibilities which may pose a significant burden on their energy level^17,18^, Lanaj et al.^19^ aptly noted that prior research has “largely overlooked the fact that leaders have limited energy reserves” (p. 1). This lack of attention can at least in part be ascribed to the belief that leaders are simply more efficient in self-regulation and are effective in pursuing the tasks they want to achieve^9^. Moreover, prior research suggests that, because those in leadership positions are more powerful, their power base itself may buffer against the negative influence of feeling depleted, which enables them to still execute tasks and act under pressure^9,10^. Because of these two reasons, an understanding has grown in the literature that—while leaders can indeed feel depleted—this does not necessarily translate into lower levels of performance.

Importantly, however, is that these prior studies did not consider fact that “leadership is an interpersonal process … in which each subordinate and leader exert mutual control and influence over one another and are mutually dependent” (^20^, p. 218). This means that leaders’ performance level not only depends on their own solitary actions, but primarily so on the help and support that they receive from their employees. This insight therefore implies that to examine whether and how depletion affects leader’s performance, we also need to examine how employees respond to the beliefs and behaviors of leaders—something that prior leadership depletion studies did not do. Indeed, although a substantive number of studies has examined how feelings of depletion may impact leaders’ behavior towards their employees (see for instance the literature on the link between leader depletion and various leadership behaviors; e.g.,^7,17,21,22^); these studies did not investigate how these employees subsequently react towards their depleted leader, and how those reciprocal actions of employees, in turn, may affect leader performance. To provide deeper insights in those broader, more distal effects of self-regulation failures, the present paper aims to investigate exactly that question. In examining this question, we rely on insights from social exchange theory and its application to self-regulation theory.

#### A social exchange approach to self-regulation theory

Earlier work into self-regulation has showed that, when people feel depleted they suffer from regulatory failures, which negatively affects their performance on a variety of cognitive, attention, intellectual, and physical performance tasks^23–25^. Schmeichel et al.^24^, for instance, reported that depleted individuals performed worse on logic, reasoning, and reading comprehension tests than non-depleted individuals. Common across these earlier studies, however, is that they started with employing an intra-individual view to understand how depletion affects performance. As such, most of them focused on individual performance levels that were achieved and measured in the context of socially isolated tasks. As research on self-regulation moved on, scholars started to examine whether depletion not only impacts people’s solitary performance, but also their responses towards others. In other words, scholars started to broaden their research questions by assuming that self-regulation failures also have important interpersonal consequences. This line of work has shown that depleted individuals are less likely to engage in positive interpersonal behaviors (like helping behaviors; see^26,27^) and more likely to fail in resisting the temptation to engage in negative interpersonal behaviors (such as lying, cheating, deception, and other unethical behaviors; see^1,17,28,29^). Finally, a recent study showed that leader depletion not only made leaders act in unethical ways, but also made their subordinates act in similar negative ways^30^.

From the above, it can thus be concluded that most prior depletion research either focused on what happens within the depleted individual him or herself (i.e., intra-individual consequences of feeling depleted) or how depletion affects the depleted individual’s reactions towards others (i.e., interpersonal consequences of feeling depleted). We argue that to fully understand the impact that a depleted state has on leader performance, we need to go one step further. Specifically, we argue that we need to examine more precisely how the intra-individual effects of depletion on a leader’s beliefs and attitudes influence how the interaction partners of the leader will respond, and how their interpersonal responses, in turn, affect leader performance. We make this argument because leadership is essentially a socially embedded construct. Or, to put it in the words of Day and Schyns (^31^, pp. 253–254), “leadership does not occur in a vacuum devoid of followers; rather, it is very much a group phenomenon where leader–follower dyads are often interdependent with each other.” This quote illustrates that leaders do not have absolute control over how well they perform; instead, they are also dependent on the contributions and the help others (such as their employees) provide them. For this reason, to provide a more complete picture of how leader depletion influences leaders’ own performance level, it is necessary to also examine how leaders’ depleted state of mind affects their interactions with their employees, and how those employees’ reciprocating reactions subsequently affect the depleted leader.

To this end, we integrate theorizing from both self-regulation theory^4,14^ and social exchange theory^32,33^ to develop a three-step sequential (serial) mediation model that articulates in detail through which pathway leader depletion can degrade their own performance level (see Fig. 1). The central tenet of social exchange theory is the norm of reciprocity^32,34^, which obligates individuals to respond positively to favorable treatment and negatively to unfavorable treatment received from others. This norm can help us to explain why depletion leads leaders to show less trust towards their employees, who will reciprocate by showing less organizational citizenship behaviors towards the leader, and how this negative reciprocity will eventually result in lower leader performance.

### Hypotheses development

#### Link between leader depletion and leaders’ trust beliefs (H1)

Trust can be defined as “the willingness of a party to be vulnerable to the actions of another party based on the expectation that the other will perform a particular action important to the trustor, irrespective of the ability to monitor or control that other party” (^35^, p. 712). Ample prior studies have found that the presence of trust is linked to a variety of positive outcomes, while its absence is associated with negative outcomes (e.g., see^35–37^). To date, however, the existing literature has hardly investigated how trust-related beliefs are linked with depletion. One of the few studies that has been conducted so far concerns the experimental research of Righetti and Finkenauer^38^, who demonstrated that people are more willing to trust someone with good rather than poor self-control. The presumed reason is that trustors anticipate that trustees with good self-control will be able to resist the temptation to exploit them for short-term advantages. Ainsworth et al.^39^ further extended this analysis by arguing that just as (and because) trustees may be tempted to exploit, trustors may be tempted to distrust them. In other words, when the chances of being exploited loom, people may be inclined to withhold their trust. In that case, self-control can override the skepticism and enable a person to take the risky step of making oneself vulnerable to others^39^. But when people are depleted, they lack the self-control that is needed to deal with possible negative outcomes and are, as a result of this, more prone to avoid risky alternatives^40^. Or, to put it in the Unger and Stahlberg’s^40^ own words, depleted individuals are simply “too exhausted to take a risk” (p. 28), and are therefore expected to also be less willing to be vulnerable towards others. Thus, insofar as trust depends on self-control, it can reasonably be assumed that people will become less trusting of others when being in a state of depletion. In line with this reasoning, Ainsworth et al.^39^ found that depleted individuals offered their counterpart less money in the context of a trust game compared to those that were not depleted. As such, this study provides a first indication that feelings of depletion may not only negatively impact other people’s trust in the depleted individual, but also the depleted individual’s trust in other people. In the present paper, we put these experimental findings in an organizational context, and argue that feeling depleted will make leaders display negative trust expectations in their relationships with employees:

**H1:** Leaders’ feelings of depletion are negatively associated with leader’s trust beliefs.

#### Link between leaders’ trust beliefs and employees’ citizenship behaviors (H2)

An important assumption of social exchange theory is that the process of trust guides reactions to subsequent exchanges^32^. In the context of our research, it can be expected that leaders’ trust beliefs will influence the way in which leaders treat their employees, which in turn is likely to affect employees’ responses. One type of employee response that exchange-based frameworks are especially relevant to concerns organizational citizenship behavior, which can be defined as “individual behavior that is discretionary, not directly or explicitly recognized by the formal reward system” (^41^, p. 4) and that contributes in supportive and positive ways to the “environment in which task performance takes place” (^42^, p. 95). Several prior studies have investigated the role that trust plays in the exhibition of such citizenship behaviors (e.g.,^43–45^). Particularly interesting in light of our research question is the study of Brower et al.^46^. These authors reported that leaders’ trust in their employees had a strong effect on employees’ display of citizenship behaviors, even beyond the effects of employees’ trust in their leader. Based on this finding, which suggest that the trust leaders display towards their employees is a critical determinant of whether these employees engage in citizenship behaviors, we predict the following:

**H2:** Leaders’ trust beliefs are positively related to employees’ display of leader-directed citizenship behaviors.

#### Link between employees’ citizenship behaviors and leader performance (H3)

So far, our model assumes that leader depletion installs negative trust beliefs, which are reciprocated by employees in such a way that they display less leader-directed citizenship behaviors. We now argue that this process will further lead to a situation where employees’ decision to show more versus less citizenship behaviors is, in turn, decisive for their leader’s performance level. That is, we expect that when employees engage in discretionary behaviors, this may benefit the performance of their leader. In line with this strain of thought, several scholars have argued that employees’ citizenship behaviors reduce the amount of time leaders have to spend on certain activities, thereby increasing the time that they can spend on other, more important tasks. Podsakoff et al. (^47^, p. 264), for instance, noted that “the more employees help each other with work-related problems, the less time a manager needs to devote to these activities; thus, freeing up the manager for more productive activities.” In a similar vein, MacKenzie et al. (^48^, p. 126) noted that “employees who exhibit ‘sportsmanship’, by not complaining to their manager about every little aspect of the job they dislike, permit the manager to ‘conserve energy’ and direct his or her attention to those aspects of the job which are important to the success of the manager’s unit.” These observations all seem to suggest that employees’ display of citizenship behaviors can indeed ease their leader’s job, and thus help him or her perform better. Conversely, when employees do not feel motivated to display citizenship behaviors, this may undermine their leader’s work progress. Based on this reasoning, we formulate the following hypothesis:

**H3***:* Employees’ display of leader-directed citizenship behaviors is positively associated with their leader’s performance level.

#### Leaders’ trust beliefs and employees’ citizenship behaviors as mediating mechanisms (H4)

The main objective of the current study is to integrate social exchange theory and self-regulation perspectives to provide a more complete picture of the pathway through which leader depletion may negatively impact leader’s own performance. Using our prior three hypotheses as a basis, we can now formulate the prediction that depleted leaders perform worse because they install negative trust beliefs that undermine employees’ citizenship behaviors. More specifically, we propose a three-step sequential (serial) mediation model in which leaders’ trust in their employees and employees’ display of leader-directed citizenship behaviors act as two key mediating mechanisms. That is, we expect that being depleted makes leaders display negative trust expectations in their relationships with employees, who may subsequently ‘pay back’ their leader by engaging in less discretionary behaviors, thereby undermining the performance of their leader. In other words, leaders’ trust beliefs and employees’ leader-directed citizenship behaviors are expected to serially mediate the impact of leader depletion on leader performance. This reasoning leads to the following serial mediation hypothesis:

**H4:** Leaders’ feelings of depletion are negatively related to their own performance via the mediating mechanisms of leaders’ trust beliefs and employees’ display of leader-directed citizenship behaviors.

#### Moderating role of belief in limited willpower (H5)

An important question to add to our research, however, is if all leaders are equally prone to display low performance levels when feeling depleted, or whether some leaders are more negatively affected by a depleted state of mind than others. Some prior studies have shown that the negative consequences of feeling depleted are constrained by people’s motivations and beliefs about self-control^49,50^. These insights suggest that there might be certain personality traits that can buffer the negative consequences of depletion. One personality trait that might be particularly relevant in the context of our research question concerns willpower beliefs. More specifically, we propose that whether the predicted detrimental effects of leader depletion take place (or not) might be contingent on the extent to which those in leadership positions believe that their willpower to exert self-control is a limited resource. Due to the reduced amount of cognitive resources, depleted individuals find it harder to exert self-control. We therefore expect that leaders’ implicit beliefs regarding their self-control as a limited resource will also impact the relationship between leader depletion and leaders’ trust in their employees. Job et al.^50^ demonstrated that individuals vary in the degree in which willpower is believed to be a limited resource. When individuals have a strong belief that their willpower is a finite resource, the negative effects of depletion are expected to be more pronounced. Stated otherwise, we expect that the negative relationship between leader depletion and leaders’ trust in their employees will be especially strong for those leaders who hold the belief that their willpower to exert self-control is limited. This reasoning results in the following moderation hypothesis:

**H5:** Belief in limited willpower moderates the relationship between leaders’ feelings of depletion and leaders’ trust beliefs in such a way that this relationship is stronger for leaders who are characterized by a high (vs. low) belief that their willpower is limited.

## Overview of the present studies

Our hypotheses were tested in a series of three studies. We started our research endeavor with two multi-source field studies (Studies 1 and 2), in which we investigated if leader depletion negatively influences leaders’ trust beliefs, if this decrease in trust subsequently negatively affects employees’ display of citizenship behaviors towards their leader, and if these lowered citizenship behaviors in turn undermine the leader’s performance level (test of **H1-H4**). Study 3 aimed to replicate the findings of Studies 1 and 2 by employing an experience sampling method research design. Study 3 additionally also investigated belief in limited willpower as a potential boundary condition of our proposed model (test of **H5**). Studies 1 and 2 were both part of a larger data collection, whereas Study 3 was conducted solely for the purpose of the present research. The data of Study 1 were analyzes using SPSS (version 27), the data of Study 2 were analyzed using Stata (release 17), and the data of Study 3 were analyzed using Mplus (version 8). The datasets of all three studies are publicly available at: https://osf.io/djnvq/.

### Study 1

Study 1 (*N* = 104 leader-subordinate dyads working in various Dutch organizations) was a cross-sectional multi-source field study. In this study, leaders provided ratings on their own depletion level and their focal subordinate’s citizenship behaviors, whereas subordinates rated their leader’s trust beliefs as well as their leader’s performance level (using two scales). Table 1 presents the means, standard deviations, and intercorrelations among the study variables.

**Table 1:** Means, standard deviations, and intercorrelations (Study 1).

Variables	*M*	*SD*	α	1	2	3	4
1. Leader depletion	2.67	1.67	.97				
2. Leaders’ trust beliefs	5.03	0.89	.85	− .50***			
3. Employees’ citizenship behaviors	4.90	0.91	.92	− .22*	.32***		
4. Leader performance (scale 1)	5.59	1.00	.93	− .49***	.60***	.43***	
5. Leader performance (scale 2)	5.42	0.96	.95	− .48***	.64***	.49***	.88***

* *p* < .05. ** *p* < .01. *** *p* < .001.

#### Results of Study 1

To test our hypotheses, we first conducted a series of linear regression analyses, of which the results are summarized in Table 2. In line with the predictions made in **H1**, we found that leader depletion was negatively related to leaders’ trust beliefs, *b* = − 0.27, *SE* = .05, *p* < .001. Moreover, leaders’ trust beliefs were positively related to employees’ display of leader-directed citizenship behaviors, *b* = 0.29, *SE* = .11, *p* = .011, a finding which corroborates **H2***.* Finally, in agreement with **H3**, we found a significant positive effect of employees’ display of leader-directed citizenship behaviors on leader performance, *b* = 0.27, *SE* = .09, *p* = .002 and *b* = 0.33, *SE* = .08, *p* < .001, for scale 1 and scale 2, respectively. We subsequently tested our proposed serial mediation hypothesis using the PROCESS macro^51^. In agreement with **H4**, the results of this analysis provided evidence for our predicted overall indirect effect of leader depletion on leader performance through leaders’ trust beliefs and employees’ citizenship behaviors (scale 1: *indirect effect* = − 0.02, CI95% = [− .062, − .004]; scale 2: *indirect effect* = − 0.03, CI95% = [− .067, − .005]).

**Table 2:** Results of the regression analyses (Study 1).

Predictors	Leaders’ trust beliefs	Employees’ citizenship behaviors	Leader performance (scale 1)	Leader performance (scale 2)
Intercept	5.75*** (0.14)	3.57*** (0.65)	2.38*** (0.64)	1.70** (0.58)
Leader depletion	− 0.27*** (0.05)	− 0.04 (0.06)	− 0.14** (0.05)	− 0.11* (0.05)
Leaders’ trust beliefs		0.29* (0.11)	0.45*** (0.10)	0.48*** (0.09)
Employees’ citizenship behaviors			0.27** (0.09)	0.33*** (0.08)
R^2^	.25	.11	.46	.52

Values reflect *b* with *SE* between parentheses. * *p* < .05. ** *p* < .01. *** *p* < .001.

### Study 2

In Study 2 (*N* = 247 leader-subordinate dyads from 25 different Taiwanese firms), we employed a longitudinal research design that consisted of three measurement moments, with a three-week interval between each moment. During the first moment, leaders rated their own level of depletion and the extent in which they trust their subordinates, whereas subordinates rated the quality of the relationship with their leader. During the second moment, leaders rated their focal subordinate’s citizenship behaviors. During the third and final moment, subordinates assessed the performance level of their leader. Table 3 presents the means, standard deviations, and intercorrelations among the study variables.

**Table 3:** Means, standard deviations, and intercorrelations (Study 2).

Variables	*M*	*SD*	α	1	2	3	4
1. Leader depletion (T1)	2.96	1.18	.86				
2. Leaders’ trust beliefs (T1)	5.87	0.76	.91	− .17**			
3. Employees’ citizenship behaviors (T2)	5.34	0.84	.93	− .10	.36***		
4. Leader performance (T3)	5.65	0.83	.95	− .10	.19**	.24***	
5. Leader-member exchange (control)	5.36	0.87	.90	.08	.12	.24***	.33***

*T1* time 1, *T2* time 2, *T3* time 3.

*p < .05. **p < .01. ***p < .001.

#### Results of Study 2

A series of multilevel regression analyses were conducted to test our hypotheses. Each dyad is nested within a firm, and by varying intercepts across firms, we take the non-independence of our observations into account. The results, which are summarized in Table 4 (Model a), illustrate that leader depletion is negatively related to leaders’ trust in their employees, *b* = − .12, *SE* = .04, *p* = .002, as such providing evidence for **H1**. Furthermore, and in agreement with **H2**, leaders’ trust in their employees was positively related to their employees’ display of leader-directed citizenship behaviors, *b* = .39, *SE* = .07, *p* < .001. In line with **H3**, a significant and positive relationship between employees’ display of leader-directed citizenship behaviors and leader performance also emerged, *b* = .22, *SE* = .06, *p* < .001. We also found evidence for our predicted serial mediation chain. That is, in support of **H4***,* the results of our mediation analysis showed that leader depletion is indirectly related to leader performance, and this by negatively influencing the extent in which leaders trust their employees which, in turn, negatively affects employees’ display of citizenship behaviors towards their leader (*indirect effect* = − 0.02, CI95% = [− .028, − .002]).

**Table 4:** Results of the regression analyses (Study 2).

Predictors	Leaders’ trust beliefs		Employees’ citizenship behaviors		Leader performance	
	Model 1a	Model 1b	Model 2a	Model 2b	Model 3a	Model 3b
Intercept	6.20*** (0.14)	5.69*** (0.31)	3.14*** (0.43)	2.32*** (0.49)	4.14*** (0.49)	3.23*** (0.51)
Leader depletion	− 0.12** (0.04)	− 0.13** (0.04)	− 0.03 (0.04)	− 0.04 (0.04)	− 0.05 (0.04)	− 0.07 (0.04)
Leaders’ trust beliefs			0.39*** (0.07)	0.36*** (0.07)	0.08 (0.07)	0.07 (0.07)
Employees’ citizenship behaviors					0.22*** (0.06)	0.15* (0.06)
Leader-member exchange		0.10 (0.05)		0.20*** (0.06)		0.26*** (0.06)
Snijders/Bosker R^2^	.02	.04	.14	.17	.06	.14
Snijders/Bosker R^2^-change		.02		.03		.08

Values reflect *b* with *SE* between parentheses. Level-1 R^2^’s are reported. ‘Model a’ refers to the model in which leader-member exchange was not included as a control, whereas ‘Model b’ refers to the model in which leader-member exchange was included as a control.

*p < .05. **p < .01. ***p < .001.

As an additional robustness test, we reran all our analyses while controlling for the quality of the leader-member exchange relationship. As shown in Table 4 (Model b), in this alternative model all significant effects remained significant, including the indirect effect of leader depletion on leader performance via leaders’ trust beliefs and employees’ citizenship behaviors (*indirect effect* = − 0.01, CI95% = [− .022, − .001]).

### Study 3

A limitation of Studies 1 and 2 is that these studies did not test our full conceptual model, which aims to also identify a potential boundary condition of our proposed serial mediation chain. Moreover, the between-person approach that we employed in our prior studies may not accurately capture the ebbs and flows of psychological states such as depletion and trust. Yet, several recent studies have shown that these variables exhibit substantial within-person variations (e.g.,^52–54^). In order to capture daily variations in the constructs of our model, in the present study we employed an experience sampling method research design (for a recent review of existing empirical research that has used this methodology to examine important leadership processes, see^55^). Note that by employing this particular design type, we were able to test if the between-person effects that we reported in Studies 1 and 2 also hold when examining within-person effects. This is particularly important given that prior research has shown that the association among variables sometimes differ depending on whether they are measured on the intra-individual or the inter-individual level (e.g., see^52^).

Participants in the present study (*N* = 50 leaders enrolled in a part-time executive MBA class at an US university) were asked to complete three surveys per day for ten consecutive work days: The morning survey measured the extent in which they felt depleted, the midday survey probed the extent in which they trusted their subordinates, and the evening survey measured the extent in which they received help from their subordinates as well as their perceived work goal progress. Additionally, we also included a trait measure of belief in limited willpower, which was administered one week before the start of the daily surveys. Table 5 displays the means, standard deviations, and intercorrelations among the study variables.

**Table 5:** Means, standard deviations, and intercorrelations (Study 3).

Variables	*M*	*SD*	α	1	2	3	4
1. Leader depletion (morning survey)	1.40	0.54	.85				
2. Leaders’ trust beliefs (midday survey)	4.17	0.34	.90	− .07			
3. Employees’ citizenship behaviors (evening survey)	2.94	0.57	.83	.04	.10		
4. Leader performance (evening survey)	4.00	0.54	.90	.05	.08	.21*	
5. Leader’s belief in limited willpower (trait)	2.79	0.99	.84	.06	− .09	− .18**	− .16**

Level-1 relationships are based on group-mean centered variables. Level-1 variables were aggregated when estimating between-individual (Level-2) correlations. *p < .05. **p < .01. ***p < .001.

#### Results of Study 3

We used multilevel modeling to account for the nested structure of our data. Specifically, the Level-1 variables were leader depletion, leaders’ trust in their employees, leaders’ experienced citizenship behaviors from employees, and leader performance, all of which could vary within individuals. The Level-2 variable was belief in limited willpower, which was specified to vary between individuals. Prior to running our analyses, we grand-mean centered this between-person variable to improve interpretability of the regression coefficients^56^. In addition, as recommended by prior literature, we group-mean centered the within-person predictors to remove between-person confounding factors^57^. Moreover, consistent with prior literature (e.g.,^18,58^), we controlled for all endogenous variables from the prior day to account for temporal changes in these variables. We further controlled for daily hours spent at work. Before testing our hypotheses, we first checked for the proportion of within-person variance in the Level-1 variables. We found substantial within-person variance in leader depletion (28.0%), leaders’ trust in their employees (66.4%), leaders’ experienced citizenship behaviors from employees (66.8%), and leader performance (40.3%), supporting the suitability of our within-person approach.

**Figure 2:**
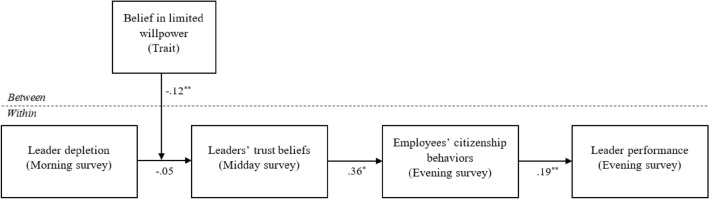
Results of multi-level path analysis (Study 3). Note. We controlled for current day working hours as well as leaders’ trust beliefs, leaders’ experienced citizenship behavior, and leader performance from the prior day. In addition, the distal effects of leader depletion on the endogenous variables were included. For clarity, these paths are not pictured in the model. * *p* < .05. ** *p* < .01. *** *p* < .001.

We subsequently conducted a multilevel path analysis. The results of this analysis are summarized in Fig. 2. **H1** predicted that leaders’ feelings of depletion are negatively associated with leaders’ perceptions of trust in their employees. This relationship, however, did not emerge as significant, *γ* = − .05, *SE* = .04, *p* = .16. **H2** predicted leaders’ trust in their employees to be positively associated with their experienced citizenship behaviors from employees. In support of this hypothesis, we found this relationship to be positive and significant, *γ* = .36, *SE* = .15, *p* = .012. **H3**, which predicted that leaders’ experienced citizenship behaviors from employees is positively associated with leader performance, also received support, *γ* = .19, *SE* = .07, *p* = .003. Finally, in line with the predictions made in **H4,** the indirect effect of leader depletion on leader performance through leaders’ trust beliefs and employees’ citizenship behaviors also turned out to be significant (*estimate of indirect effect* = − .004, CI95% = [− .0125, − .0001]).

**Figure 3:**
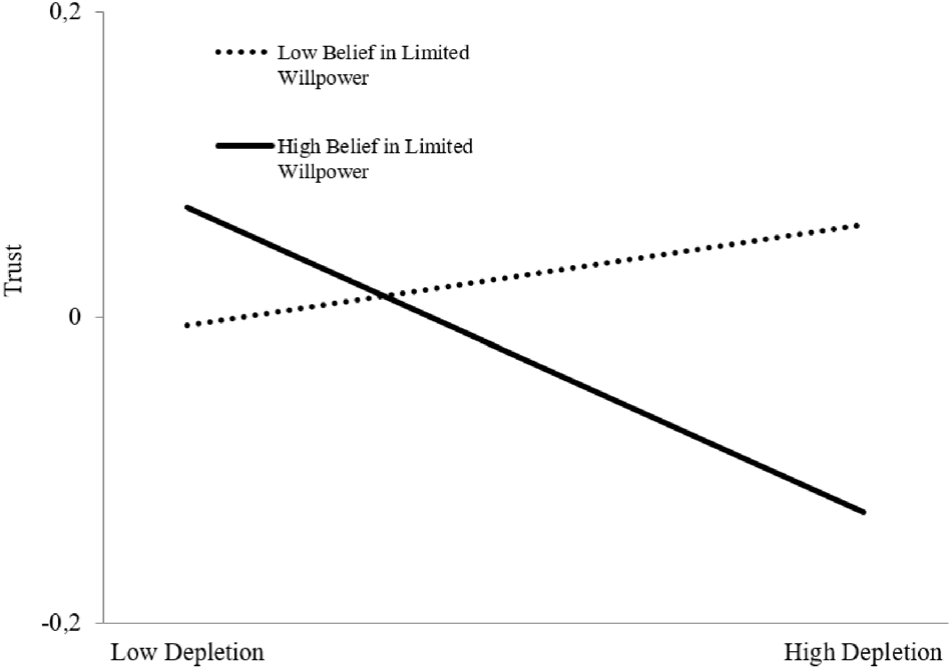
Cross-level interaction of leaders’ trait belief in limited willpower and leader depletion predicting leaders’ trust beliefs (Study 3). Note The relationship between leaders’ feelings of depletion and leaders’ trust in their employees was significant (*Est.* =  − .17, *SE* = .06, *p* = .006) at *high* levels of belief in limited willpower, but not significant (*Est.* = .07, *SE* = .05, *p* = .183) at *low* levels of belief in limited willpower.

To test our moderation hypothesis, we subsequently examined if and how the relationship between leader depletion and leaders’ trust in their employees is influenced by leaders’ belief that their willpower is a limited resource. This cross-level interaction was tested by having belief in limited willpower as a Level-2 predictor of the intercept and slope for the Level-1 relationship between leaders’ feelings of depletion and leaders’ trust in their employees. This analysis revealed that the cross-level interaction of leader belief in limited willpower and leader depletion predicting leaders’ trust beliefs was significant, *γ* = − .12, *SE* = .05, *p* = .009. To further investigate the nature of this interaction, in Fig. 3 we plotted the relationship between leaders’ feelings of depletion and leaders’ trust in their employees at high and low conditional values of leader belief in limited willpower (+ 1 SD and − 1 SD; see 56). In line with **H5**, we found that when belief in limited willpower was *high*, the relationship between leader depletion and leaders’ trust in their employees was strong and negative (*simple slope estimate* = − .17, *SE* = .06, *p* = .006). This relationship, however, was not significant when belief in limited willpower was *low* (*simple slope estimate* = .07, *SE* = .05, *p* = . 183).

To assess the conditional indirect effects where the proposed indirect effect is moderated by belief in limited willpower, we additionally also conducted a Monte Carlo simulation with 20,000 replications to obtain bias-corrected confidence intervals around the estimates^59^. These conditional indirect effects of our full model indicate that the indirect effect of leader depletion on leader performance via leaders’ trust in their employees and leaders’ experience of citizenship behaviors from employees was significant and negative when belief in limited willpower was *high* (*estimate* = − .012, CI95% = [− .037, − .003]), whereas it was non-significant when belief in limited willpower was *low* (*estimate* = .005, CI95% = [− .001, .023]). Note that these latter findings are in line with the simple slopes results (see above), as well as with our full conceptual model.

## Discussion

Prior depletion research has mainly looked at how the state of depletion influences leaders’ performances on tasks that are segregated from the inherent social role that they occupy within their organization. As such, these studies have neglected the fact that many leadership tasks are interdependent in nature and thus require cooperation with others, most notably their employees. In line with this strain of thought, we proposed that leader depletion indirectly impact leaders’ own performance level via the mediating mechanisms of leaders’ trust beliefs and employees’ display of leader-directed citizenship behaviors. The results of two multi-source field studies (Studies 1 and 2) provided robust evidence for our predicted serial mediation chain. Using an experience sampling methodology, Study 3 extended these findings by showing that leaders who have a strong (vs. a weak) belief that their willpower is limited were more strongly affected by the negative effects of feeling depleted. Yet, unlike in Studies 1 and 2, in Study 3 we did not observe a significant main effect of leader depletion on leaders’ trust beliefs. A possible explanation for this discrepancy may be that effects might be smaller on the daily level, as opposed to the cumulative effects in Studies 1 and 2.

### Theoretical contributions

A number of studies has revealed that the general idea behind ego depletion theory is that the capacity to exert self-control is a limited resource that is depleted after exertion^4^. This idea seems to be especially at place for those in positions where they face high workloads, have many responsibilities, and steer collaborative challenges, which usually are leadership positions^16,19^. In fact, examining the role that depletion plays especially among leaders is an interesting and important task given the fact that leadership within organizations is an interdependent activity which makes that multiple parties are affected.

Yet, as we mentioned earlier, prior depletion research has focused almost exclusively on how depletion influences outcomes of the depleted individual (e.g.,^23,24^) or how depleted individuals behave towards others (e.g.,^21,22^), but has not extended these perspectives. An important theoretical contribution of our work is that our studies go beyond this approach by also testing the broader, more distal implications of this negative leader state. Specifically, in our studies we do zoom in on the outcomes and beliefs of the depleted individual (i.e., showing low trust beliefs towards others), but add the perspective where we examine how those beliefs affect others that have an interdependent relationship with the leader (in our case their employees) and how the reciprocal behaviors of those others affect the performance level of the leader in return.

In light of this perspective, it must be noted that several prior studies on the consequences of self-regulation failures have found that feelings of depletion can indeed hinder performance (see^60^, for an overview). Yet, an important implicit assumption of self-regulation theory is that the effects of depletion on performance are fleeting, meaning that once depleted resources are replenished performance should almost immediately be restored. This assumption makes most sense when investigating depletion effects in the context of socially isolated tasks, but in real-life settings, where social dynamics are at play, the consequences of leader depletion are likely to be more insidious. It is, in other words, possible that in the workplace leader depletion also has more lingering negative effects, which would imply that its negative impact may persist even when the source of depleting is being removed.

Our findings provide some indication that leader depletion may indeed have such persisting negative effects. The results of our second study, for instance, showed that leader depletion was negatively linked with leader performance via the mediating mechanisms of leaders’ trust beliefs and employees’ leader-directed citizenship behaviors, and this even though leader performance was measured several weeks after the measurement of leader depletion. Because low trust as a result of earlier depleted states of the leader cannot easily be restored (see for instance the trust repair literature; e.g.,^61,62^), it is possible that the leader’s performance level remains low—even when he or she no longer feels depleted. We therefore strongly encourage future research in this domain to further unravel the potential long-term negative consequences that leader depletion might have for organizational functioning.

### Practical implications

Besides several theoretical contributions, the present research also offers some important practical insights for organizations and its members. Being in a leadership position comes with heavy workloads and with numerous choices and decisions that need to be made each day. From a managerial perspective, we believe that it is crucial for organizations to be aware that overloading their leaders in this respect may bring unwanted costs. Of course, creating awareness itself is not enough. Organizations should also try to actively prevent their leaders from becoming depleted. In this light, several studies, for example, have reported that brief mindfulness interventions can decrease feelings of exhaustion (e.g.,^63–65^). Organizations who aim to counteract the detrimental effects of leader depletion are therefore advised to make such interventions available to their leaders.

Moreover, leaders themselves should also understand that, whenever they face important tasks, their cognitive state can affect their performance level; thus, it is necessary to schedule such tasks carefully. Because of this, tasks that have important implications and/or that might significantly affect others—like strategic actions and ethical decisions—should preferably be executed by leaders when they are well rested (e.g., in the morning, after a good night of sleep) rather than when they are tired and fatigued (e.g., late in the day, or when deprived of sleep), because rest (and especially sleep) can replenish their cognitive resources (see^60,66^).

Critically, however, our findings also convey a more optimistic message, by illustrating that not all leaders are equally prone to display low performance levels when their resources are depleted. More specifically, the results of our third study, which set out to identify a potential boundary condition of our proposed sequential mediation model, revealed that for those leaders who believe that their willpower to exert self-control is unlimited (i.e., low instead of high belief in limited resources) there was no indirect negative effect of leader depletion on leader performance via leaders’ trust in employees and employees’ leader-directed citizenship behaviors, indicating that these particular leaders may actually be immune to the negative effects of feeling depleted.

We believe that this latter finding is particularly important given that “willpower is not exclusively determined by ‘nature’, but it is also something that can be trained” (^67^, p. 263). Prior willpower training interventions have indeed revealed that—similar to a muscle—willpower can be strengthened with practice^68^. Interestingly, however, is that several studies have even shown that training individual acts of self-control in any domain (from improving your posture to watching your finances) can increase ‘overall’ willpower^69,70^. Organizations are therefore encouraged to also provide systematic training programs that can help leaders to enhance their willpower, as such training programs might be helpful to mitigate the potential negative effects of leader depletion.

### Strengths, limitations, and future research

A strength of this work lies in the fact that our studies consisted of both between-person (Studies 1 and 2) and within-person assessments (Study 3) of our core concepts. With respect to our measure of leader performance (our focal outcome variable), subordinates’ peer-ratings (Studies 1 and 2) ware complemented with leaders’ self-ratings (Study 3). Moreover, to increase the cross-cultural generalizability of the reported findings, our studies were conducted among participants from three different continents, those being: Europe (Study 1), Asia (Study 2), and North-America (Study 3). Despite these differences, our studies revealed very similar results, as such promoting our confidence in the robustness of the reported findings.

Yet, no research is without its limitations, and our paper is no exception. A first and foremost limitation of our work is that, although we employed a longitudinal approach in our second study, we cannot infer causality from any of our studies, as we did not manipulate variables or use random assignment techniques. Instead, in each of our studies we tested our predictions in existing leader-subordinate relationships using survey data. In this regard, both Spencer, Zanna, and Fong^71^ and Bullock, Green, and Ha^72^ have argued that only so-called “experimental-causal-chain” designs, in which both mediators are sequentially manipulated in separate experiments, can resolve issues related to inferring causality from statistical analysis. Hence, we strongly encourage future researchers to implement such designs, in order to further disentangle the causal roles that leaders’ trust beliefs and employees’ citizenship behaviors play in the relation between leader depletion and leader performance.

Another issue that must be mentioned here is that, due to the nature of our data collection, we do not know what caused leaders to feel depleted in our studies. A recent study by Zhang et al.^73^ illustrated that depletion can either be internally or externally imposed. Internally imposed depletion is depletion due to personal factors, whereas externally imposed depletion is depletion due to situational factors. In the context of our work, we expect that the source of depletion may influence whether an increase in depletion translates into a decrease in trust or not. It is, for instance, possible that depleted leaders only trust their employees less when they see these employees as the cause of their depletion (i.e., externally imposed depletion), but not when they see themselves as being responsible (i.e., internally imposed depletion). This assumption, which can possibly also help to explain why we did not find a significant main effect of leader depletion on leaders’ trust beliefs in our third study, needs to be further examined in future research.

Our research is a first step in mapping out through which pathway leader depletion may negatively influence leader’s own performance. However, to get a more complete picture of the link between depletion and performance, future research might also want to investigate how depletion spreads and fluctuates within teams. Finally, our research illustrates that whether leader depletion negatively impacts leader performance depends on the extent to which those in leadership positions believe that their willpower to exert self-control is a limited resource. It is, however, important to note that, besides willpower beliefs, other individual difference variables might exist that also moderate the impact that leader depletion has on leaders’ trust beliefs. Future research in this domain is therefore encouraged to further investigate which other leader traits can buffer the negative effects of feeling depleted.

## Methods

Given that our studies were conducted according to the ethical rules presented in the General Ethical Protocol for Scientific Research of the Faculty of Psychology and Educational Sciences of Ghent University (https://www.ugent.be/pp/en/research/ec/general_ethical_protocol_fppw.pdf), where the first author is affiliated, no formal ethical approval was required. Informed consent was obtained from every participants.

### Study 1

#### Sample and procedure

In Study 1, we used Flycatcher, a professionally managed panel established by the University of Maastricht, to reach a broad sample of the working population in the Netherlands. The Flycatcher panel has the ISO-26362 certification for access panels (i.e., it meets the qualitative ISO requirements for social scientific research, market research, and opinion polls). In return for their voluntary involvement in completing questionnaires, panel members receive a small reward in the form of points, which they can collect and eventually convert into a voucher of their choice.

We only invited participants who have a leadership position at work. Leaders were asked to identify one of their subordinates to answer a brief survey. By entering their subordinate’s email address, a message was automatically generated that asked this person to participate in the study. Each participant received a unique identification number to ensure anonymity and to allow the proper matching of focal subordinate and leader data. This procedure generated a final sample of 104 unique leader**-**subordinate dyads. The mean age of the leaders was 42.64 years (*SD* = 9.59), 62.5% of which were male. The mean age of the matched group of subordinates was 36.68 years (*SD* = 10.42), 56.7% of which were male.

Several steps were taken to optimize data validity and to ensure that survey responses corresponded to the correct organizational role (i.e., the leader or the subordinate role). First of all, when introducing the study, we noted to all participants that integrity is crucial in the scientific process and, therefore, compliance with the instructions is necessary. Moreover, it was also clarified that it was necessary and important that the leader and the corresponding subordinate filled in the correct survey. When participants submitted their online survey, time stamps and IP addresses were recorded to ensure that the surveys were submitted at different times and with different IP addresses. No irregularities in the responses were found.

#### Measures

During the study, leaders provided ratings on their own depletion level and their subordinates’ citizenship behaviors, whereas subordinates rated their leader’s trust beliefs as well as their leader’s performance level. All items were answered on seven-point Likert scales*.* The full item lists are included in the Supplementary Material file.

##### Leader depletion

Five items adapted from Ciarocco et al. (^74^; see also ^18^) probed the extent in which leaders feel depleted. A sample item is: “I feel mentally exhausted” (1 = *strongly disagree*, 7 = *strongly agree*). This scale was answered by the leader him or herself (Cronbach’s alpha = .97).

##### Leaders’ trust beliefs

Leaders’ trust beliefs were measured using an eight-item scale, of which the items are based on the NEO-PI-R^75^. A sample item is: “My supervisor thinks that most of the people he or she deals with are honest and trustworthy” (1 = *strongly disagree*, 7 = *strongly agree*). This scale was answered by the subordinate (Cronbach’s alpha = .85).

##### Employees’ citizenship behaviors

To measure the extent in which employees engage in leader-directed citizenships behaviors, we used an eight-item scale of which the items are based on the scale of Lee and Allen^76^. A sample item is: “My subordinates defend me when other employees criticize me” (1 = *never*, 7 = *always*). The leader answered this scale (Cronbach’s alpha = .92).

##### Leader performance

Finally, we employed two different performance scales to measure subordinates’ perceptions of their leader’s performance. The first scale is a six-item scale of which the items are based on the six in-role performance criteria of De Cuyper et al. (^77^; also see ^78^). A sample item is: “To what extent do you expect that your supervisor is able to achieve his or her objectives” (1 = *not at all*, 7 = *very much*). The second scale is a ten-item scale of which the items are adapted from the performance scale of Wright et al.^79^. A sample item is: “I would never be disappointed in the quality of the performance that my supervisor delivers” (1 = *strongly disagree*, 7 = *strongly agree*). Both these performance scales were answered by the subordinate (Cronbach’s alpha = .93 and .95, for scale 1 and scale 2, respectively).

### Study 2

#### Sample and procedure

Twenty-eight firms in Taiwan were contacted by the research team with the question to participate in our study. These firms operated in manufacturing, education, finance, service, health care, and retail sectors. A variety of firms were chosen to increase the generalizability of our findings. We first visited members of the top management team of each firm, and successfully received their consent and support for our study. During the study, we worked closely with the corresponding HR department of each firm so that the surveys could easily be distributed to the leader-subordinate dyads, which were randomly selected from different teams by the HR department of each firm. To ensure high quality responses, we guaranteed the HR departments that integrity and honesty would be highly emphasized during the data collection process.

The data collection was organized into three separate phases, which were conducted in three-week intervals. With the assistance of the HR departments of the 28 contacted firms, we initially reached out to a total of 362 matched leader-subordinate dyads. During the first data collection phase (Time 1), we sent a survey containing measures of demographics to all participants. At this time point, leaders were also asked to rate their own level of depletion as well as the extent in which they trust their subordinates. We received responses from 279 matched leader-subordinate dyads. During the second data collection phase (Time 2), leaders were asked to rate their focal subordinate’s citizenship behaviors. In total, 253 leaders of the matched leader-subordinate dyads successfully completed the survey during the second data collection phase. During the third and final data collection phase (Time 3), we asked each subordinate from the 253 matched leader-subordinate dyads to assess the performance level of their leader and received responses from all of the subordinates. Note that this temporal separation of our study variables is consistent with the causal order of our proposed sequential mediation model.

A total of six participants did not fully respond to all the items from our study variables. Because of this, our final sample consisted of 247 unique leader-subordinate dyads from 25 different firms (i.e., 89% of the dyads who completed the first data collection phase); with the average number of leader-subordinate dyads from each firm being 9.88 (range = 3–35) and with each leader-subordinate dyad coming from of a different team. The mean age of the leaders was 42.77 years (*SD* = 9.27), 38.5% of which were male. The mean age of the matched group of subordinates was 33.33 years (*SD* = 7.86), 32.8% of which were male.

#### Measures

With exception of the leader-member exchange items, all items were answered on seven-point Likert scales ranging from (1) *strongly disagree* to (7) *strongly agree*. The full item lists are included in the Supplementary Material file.

##### Leader depletion (Time 1)

To measure the extent in which leaders feel depleted, we again used five-items which are adapted from Ciarocco et al.’s^74^ scale. A sample item is: “I have felt drained.” The leader answered this scale during the first data collection phase (Cronbach’s alpha = .86).

##### Leaders’ trust beliefs (Time 1)

During the first data collection phase, leaders’ trust in their employees was also measured using a five-item scale developed by De Jong and Elfring^80^. A sample item is: “I trust my team members.” So, contrary to Study 1, in the present study these trust belief items specifically referred to the leader’s trust in his or her subordinates. Moreover, in the present study the leader (instead of the subordinate) answered this measure (Cronbach’s alpha = .91).

##### Employees’ citizenship behaviors (Time 2)

Leaders were also asked to rate the extent in which their focal employee engages in leader-directed citizenships behaviors. Towards this end, we again administered an eight-item scale of which the items are based on the scale of Lee and Allen^76^. A sample item is: “My subordinate willingly gives his or her time to help me when I have work-related problems.” This scale was administered during the second data collection phase (Cronbach’s alpha = .93).

##### Leader performance (Time 3)

During the third and final data collection phase, subordinates assessed their leader’s performance with the job performance scale developed by Podsakoff and McKenzie (^81^; cited and used by ^82^). This scale was adapted so that it measures the extent to which subordinates agree with five statements about the quality and the quantity of their leader’s performance. A sample item of this scale is: “My supervisor fulfils all responsibilities required by his or her job” (Cronbach’s alpha = .95).

##### Leader-member exchange (control variable)

Given that prior research has shown strong relationships between leader-member exchange and many of the variables that are included in our model (for examples, see^83–85^), we measured the quality of the leader-member exchange relationship as a control variable. To this end, subordinates were asked to complete a seven-item scale of which the items are based on the leader-member exchange (LMX) scale of Graen and Uhl-Bien (^86^; also see^87^). They answered this scale during the first data collection phase.

### Study 3

#### Sample and Procedure

The sample for our experience sampling study consisted of 52 leaders enrolled in a part-time executive MBA class at a large midwestern university in the United States. They worked in a large variety of industries, including manufacturing, information technology, healthcare, accounting, banking or finance, engineering, and transport or logistics. We collected data over a two-week period using a series of daily surveys that were delivered online via email. One week before we began administering the daily surveys, leaders completed a one-time survey that assessed individual differences in belief in limited willpower as well as demographic information. Afterwards, we administered daily assessments for ten consecutive workdays. Similar to prior experience sampling research in the management literature (e.g.,^19,21^), we chose a two-week period. During the two weeks of the study (including only workdays), participants were asked to complete three surveys per day. In line with the causal order of our hypothesized mediation model, the morning survey (sent at the start of the workday) measured leaders’ feelings of depletion. The midday survey (sent in the middle of the workday) assessed leaders’ trust in their employees. Finally, the evening survey (sent at the end of the workday) captured leaders’ experienced citizenship behaviors from employees as well as leaders’ perceived work goal progress.

After screening out missing responses and accounting for days where leaders did not report interacting with any employee, our final dataset was composed of 365 daily matched observations from 50 leaders (73% of all possible daily observations). This sample was 67.7% male and the majority (77.0%) of the leaders were between 31 and 60 years of age. They reported a mean organizational tenure of 6.26 years (*SD* = 6.24) and managed an average of 4.92 employees (*SD* = 7.62).

#### Measures

The Supplementary Material file provides the full item lists of our between-person measure (i.e., belief in limited willpower) and our four within-person measures (i.e., leader depletion, leaders’ trust beliefs, employees’ citizenship behaviors, and leader performance).

##### Leaders’ belief in limited willpower (trait)

One week before we started the daily surveys, belief in limited willpower was measured using a scale developed by Job et al.^50^. This scale consists of three items measuring the extent to which leaders agreed with their willpower beliefs being limited. An example item is: “Strenuous mental activity always exhausts your resources, which you need to refuel afterwards (e.g., through breaks, doing nothing, watching television, eating)” (1 = *strongly disagree*, 5 = *strongly agree;* Cronbach’s alpha = .84).

##### Daily leader depletion (morning survey)

To assess daily fluctuations in leader depletion, we adapted three items from scales representing the content domain of depletion^74,88^. A sample items is: “Right now, I feel mentally drained”. For two consecutive weeks, leaders answered this scale daily during the morning survey. Leaders were asked to report the extent in which they felt these states at that specific moment (1 = *not at all,* 5 = *to a very large extent;* Cronbach’s alpha = .85).

##### Daily leaders’ trust beliefs (midday survey)

Daily fluctuations in leaders’ trust in their employees were measured using the five-item scale by De Jong and Elfring^80^, which was also used in Study 2. A sample item of this scale is: “Right now, I am confident that my subordinates will keep me informed about issues that concern my work.” Leaders answered this scale daily during the midday survey, and they were asked to report the extent to which they trusted their subordinates at that particular moment (1 = *not at all, 5* = *to a very large extent;* Cronbach’s alpha = .90).

##### Daily employees’ citizenship behaviors (evening survey)

We used the six-item scale by Dalal et al.^89^ to capture daily fluctuations in the extent in which leaders received helping behavior from their employees. Leaders answered this scale each day during the evening survey. An example item is: “So far today, my subordinates have gone out of their way to be nice to me” (1 = *not at all, 5* = *to a very large extent;* Cronbach’s alpha = .83).

##### Daily leader performance (evening survey)

Finally, daily fluctuations in leaders’ perceptions of their own work goal progress were assessed using three items developed by Koopman et al.^90^. These items, which were also administered daily during the evening survey, captured the leader’s perceived goal progress so far during that day. An example item is: “Today, I have moved forward with my work progress” (1 = *strongly disagree*, 5 = *strongly agree;* Cronbach’s alpha = .90).

### Ethical approval

The research was conducted according to the ethical rules presented in the General Ethical Protocol of the Faculty of Psychology and Educational Sciences of Ghent University (https://www.ugent.be/pp/en/research/ec/general_ethical_protocol_fppw.pdf). Informed consent was obtained from all participants.

## Supplementary Information

Supplementary Information.

## Data Availability

The data that are reported in the present manuscript are made publicly available and can be openly accessed through Open Science Framework: https://osf.io/djnvq/. The data and data analysis scripts can also be requested from the first author: tessa.haesevoets@ugent.be.
