# Supplementary material for: The impact of leader depletion on leader performance: the mediating role of leaders’ trust beliefs and employees’ citizenship behaviors
Source: Sci Rep. 2022 Nov 30;12:20676. doi: 10.1038/s41598-022-24882-3 (PMC9712642; doi:10.1038/s41598-022-24882-3)
Supplement: Supplementary file 1 — Supplementary Information. [file 41598_2022_24882_MOESM1_ESM.docx]

**SUPPLEMENTARY MATERIAL FILE**

**Full Item Lists of Studies 1-3**

**Study 1**

***Leader depletion (self-rated by the leader)***

1. I feel mentally exhausted.

2. Right now, it would take a lot of effort for me to concentrate on something.

3. I feel drained.

4. My mind feels unfocused right now.

5. My mental energy is running low.

***Leaders’ trust beliefs (peer-rated by the subordinate)***

1. My supervisor assumes that people are cynical and skeptical about the intentions of others.

2. My supervisor believes that most people are essentially of good will.

3. My supervisor believes that most people will use you if you give them the chance.

4. My supervisor thinks that most of the people he or she deals with are honest and trustworthy.

5. If someone does something nice for my supervisor, he becomes suspicious.

6. My supervisor’s first reaction is to trust people.

7. My supervisor usually thinks the best of people.

8. My supervisor is quite confident in human nature.

***Employees’ citizenship behaviors (peer-rated by the leader)***

1. My subordinates defend my me when other employees criticize me.

2. My subordinates show genuine pride in me when discussing me with others within my organization.

3. My subordinates offer ideas to me to make me more effective.

4. My subordinates express loyalty towards me.

5. My subordinates take action to protect me from potential problems.

6. My subordinates demonstrate concern about my image.

7. My subordinates offer to do things outside their usual job responsibilities to help me when I am absent.

8. My subordinates show a genuine concern and courtesy towards me even under the most trying business or personal situations.

***Leader performance (peer-rated by the subordinate)***

*Scale 1 (De Cuyper et al.’s scale)*

1. To what extent do you expect that your supervisor is able to make decisions?

2. To what extent do you expect that your supervisor is able to perform without mistakes?

3. To what extent do you expect that your supervisor is able to devote him or herself to work?

4. To what extent do you expect that your supervisor is able to achieve his or her objectives?

5. To what extent do you expect that your supervisor is able to take initiatives?

6. To what extent do you expect that your supervisor is able to take responsibility?

*Scale 2 (Wright et al.’s scale)*

1. My supervisor exhibits an underlying concern for doing things or tasks better, for improving situations.

2. My supervisor exhibits zeal about the job and a consequent willingness to work hard and energetically.

3. My supervisor exhibits a willingness to go beyond what the situation requires and to act before being asked.

4. My supervisor exhibits an ability to see the whole, its parts and relations, and use this to set priorities, plan, anticipate, and evaluate.

5. My supervisor gets things done on time.

6. I would never be disappointed in the quality of the performance that my supervisor delivers.

7. My supervisor shows exemplary work habits.

8. My supervisor continues to be productive even if I would not be around.

9. My supervisor does not have to be checked up on by me.

10. My supervisor gets along well with his/her co-workers.

**Study 2**

***Leader depletion (Time 1; self-rated by the leader)***

1. I have felt drained.

2. My mind has felt unfocused over the past four weeks.

3. Over the past month, it has taken a lot of effort for me to concentrate on something.

4. I have not been able to absorb any information.

5. I have felt like my willpower is gone.

***Leaders’ trust beliefs (Time 1; self-rated by the leader)***

1. I am able to count on my team members for help if I have difficulties with my job.

2. I am confident that my team members will take my interests into account when making work-related decisions.

3. I am confident that that my team members will keep me informed about issues that concern my work.

4. I can rely on my team members to keep their word.

5. I trust my team members.

***Employees’ citizenship behaviors (Time 2; peer-rated by the leader)***

1. My subordinate helps me when I am absent.

2. My subordinate willingly gives his or her time to help me when I have work-related problems.

3. My subordinate adjusts his or her work schedule to accommodate my requests for time off.

4. My subordinate goes out of the way to make me feel welcome in the work group.

5. My subordinate shows genuine concern and courtesy toward me, even under the most trying business or personal situations.

6. My subordinate gives up time to help me when I have work or nonwork problems.

7. My subordinate assists me with my duties.

8. My subordinate shares personal property with me to help my work.

***Leader performance (Time 3; peer-rated by the subordinate)***

1. My supervisor always completes the duties specified in his or her job description.

2. My supervisor meets all the formal performance requirements of his or her job.

3. My supervisor fulfils all responsibilities required by his or her job.

4. My supervisor never neglects aspects of the job that he or she is obligated to perform.

5. My supervisor successfully performs essential duties.

***Leader-member exchange (Control; rated by the subordinate)***

1. Do you usually feel that you know where you stand and do you usually know how satisfied your supervisor is with what you do?

2. How well do you feel that your supervisor understands your problems and needs?

3. How well do you feel that your supervisor recognizes your potential?

4. Regardless of how much formal authority your supervisor has built into his or her position, what are the chances that he or she would be personally inclined to use power to help you solve problems in your work?

5. Regardless of the amount of formal authority your supervisor has, to what extent can you count on him or her to “bail you out” at his or her expense when you really need it?

6. I have enough confidence in my supervisor that I would defend and justify his or her decisions if he or she were not present to do so?

7. How would you characterize your working relationship with your supervisor?

**Study 3**

***Leaders’ belief in limited willpower (Trait; self-rated by the leader)***

1. Strenuous mental activity always exhausts your resources, which you need to refuel afterwards (e.g. through breaks, doing nothing, watching television, eating).

2. After a strenuous mental activity, your energy is usually depleted and you must rest to get it refueled again.

3. When you have completed a strenuous mental activity, you cannot start immediately with the same concentration because you have to recover your mental energy again.

***Leader depletion (Morning survey; self-rated by the leader)***

1. Right now, I feel mentally drained.

2. Right now, my mental energy is running low.

3. Right now, I feel like my willpower is gone.

***Leaders’ trust beliefs (Midday survey; self-rated by the leader)***

1. Right now, I am able to count on my subordinates for help if I have difficulties with my job.

2. Right now, I am confident that my subordinates will take my interests into account when making work-related decisions.

3. Right now, I am confident that my subordinates will keep me informed about issues that concern my work.

4. Right now, I can rely on my subordinates to keep their word.

5. Right now, I trust my subordinates.

***Employees’ citizenship behaviors (Evening survey; peer-rated by the leader)***

1. So far today, my subordinates have gone out of their way to be nice to me.

2. So far today, my subordinates have tried to help me.

3. So far today, my subordinates have defended my opinion or suggestion.

4. So far today, my subordinates have gone out of their way to include me in a conversation.

5. So far today, my subordinates have tried to be available to me.

6. So far today, my subordinates have spoken highly about me to others.

***Leader performance (Evening survey; self-rated by the leader)***

1. Today, I have made good progress on my work goals.

2. Today, I had a productive day today in regards to my work progress.

3. Today, I have moved forward with my work progress.
